# Feasibility of Frequency-Modulated Wireless Transmission for a Multi-Purpose MEMS-Based Accelerometer

**DOI:** 10.3390/s140916563

**Published:** 2014-09-05

**Authors:** Alessandro Sabato, Maria Q. Feng

**Affiliations:** 1 Dipartimento di Ingegneria Meccanica, Energetica e Gestionale (DIMEG), Università della Calabria, Via P. Bucci 46C, Rende (CS), Italy; 2 Civil Engineering & Engineering Mechanics Department (CEEM), Columbia University, 500 W 120th St., New York, NY 10027, USA; E-Mail: mqf2101@columbia.edu

**Keywords:** acceleration measurement, MEMS sensor, prototype, structural health monitoring, vibration measurement, voltage to frequency conversion, wireless accelerometer

## Abstract

Recent advances in the Micro Electro-Mechanical System (MEMS) technology have made wireless MEMS accelerometers an attractive tool for Structural Health Monitoring (SHM) of civil engineering structures. To date, sensors' low sensitivity and accuracy—especially at very low frequencies—have imposed serious limitations for their application in monitoring large-sized structures. Conventionally, the MEMS sensor's analog signals are converted to digital signals before radio-frequency (RF) wireless transmission. The conversion can cause a low sensitivity to the important low-frequency and low-amplitude signals. To overcome this difficulty, the authors have developed a MEMS accelerometer system, which converts the sensor output voltage to a frequency-modulated signal before RF transmission. This is achieved by using a Voltage to Frequency Conversion (V/F) instead of the conventional Analog to Digital Conversion (ADC). In this paper, a prototype MEMS accelerometer system is presented, which consists of a transmitter and receiver circuit boards. The former is equipped with a MEMS accelerometer, a V/F converter and a wireless RF transmitter, while the latter contains an RF receiver and a F/V converter for demodulating the signal. The efficacy of the MEMS accelerometer system in measuring low-frequency and low-amplitude dynamic responses is demonstrated through extensive laboratory tests and experiments on a flow-loop pipeline.

## 1. Introduction

In recent decades, a great deal of attention has been given to the possibility of measuring physical quantities using Micro Electro-Mechanical System (MEMS) sensors and transmitting data wirelessly. In general, the wireless transmission of sensor signals, compared to its wired counterpart, is preferable due to its absence of triboelectric noise and elimination of the requirement for cumbersome cabling. The fast growth of the wireless technology piezoelectric based applications, such as Electro Mechanical Impedance (EMI) and Micro electro-mechanical systems, is changing the way civil structures and mechanical systems are monitored, controlled, and maintained. While EMI technique results in high frequency signals (e.g., [1–6]), MEMS technique results in low frequency signals. networks for Structural Health Monitoring (SHM) purposes (e.g., [7–12]).

The SHM applications require the MEMS accelerometers to be accurate for measuring a wide range of structural vibration: from ambient vibration (in the order of 10^−2^ ms^−2^) to severe earthquake (in the order of 10 ms^−2^). In addition, natural frequencies in large civil engineering structures including bridges and buildings are generally in the order of 10^−1^ to 10^1^ Hz [13]. Therefore, sensor systems able to accurately measure such low-frequency signals are required. However, currently available low-cost wireless sensor boards mounted with MEMS accelerometers are characterized by low frequency sensitivity and high noise-density, and, thus, are suited for measuring only high-amplitude vibration (nearly 10^−1^ m·s^−2^) [14].

Besides the choice of the accelerometer, the performance of MEMS sensor systems also depends on the Analog to Digital Conversion (ADC) embedded in the board itself [15]. Improvements made in MEMS accelerometers and ADCs [16] have resulted in better wireless signal quality. One example is a high-sensitivity (0.20 V·m^−1^·s^2^) and relatively low noise-density (49 μms^−2^·Hz^−0.5^) single-axis accelerometer [17]. Nevertheless, studies are more focused on improving on-board computational tasks and have shown that the board, on which that accelerometer is mounted, allows measurement of vibration having amplitude higher than a certain value and frequencies lower than approximately 2 Hz [18,19].

Aiming at cost-effective and accurate measurement of low-frequency, low-amplitude vibration, a new wireless MEMS accelerometer system is developed in this study. It is demonstrated that by employing the Voltage to Frequency Conversion V/F (instead of ADC), it is possible to achieve performances comparable to those of traditional wired accelerometers when low-amplitude (in the order of 10^−2^ m·s^−2^) and low-frequency (in the order of 10^−1^ Hz) vibration are measured. It even if a less sensitive MEMS accelerometer (0.12 V·m^−1^·s^2^) is used. This paper presents the design of a prototype of the MEMS accelerometer system and its experimental evaluations. It is important to point out that this prototype has a lower cost if compared to other SHM systems [20] or to other equivalent-sensitivity wired accelerometers [21].

## 2. Design of the Accelerometer Board

**Figure 1.**
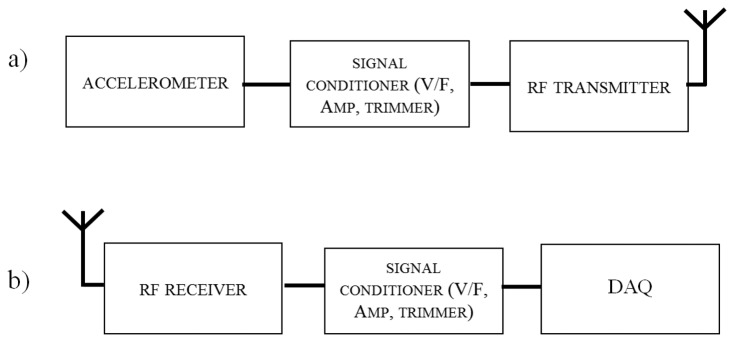
(**a**) Transmitter board block diagram. (**b**) Receiver board block diagram.

The wireless MEMS accelerometer system consists of a transmitter board and a receiver board. Figure 1 shows the block diagrams of the proposed system.

The transmitter board is installed with a selected sensing element: Colibrys SiFlex 1600SN.A (SF1600). It is a single-axis MEMS accelerometer with a low noise-density, a wide dynamic range and frequency response. The SF1600 operates with a power supply voltage that can range from ±6 V to ±15 V with a typical current consumption of 11 mA at ±6 V. The receiver board, instead, is equipped with a section for reconverting the received signal in tension values and transmits it to a data acquisition (DAQ) device.

Table 1 summarizes the main features of the accelerometer determined at +20 °C and with 15 V DC power supply [22].

From the block diagram in Figure 1, it is important to notice that an on-board microcontroller is not installed. Raw data have to be downloaded in an external laptop for being post-processed. Even if one of the basic ideas of SHM is to transmit essential information only; the authors move in the opposite direction with this study. Many other engineering sectors need several data to determine the occurrence of possible critical scenarios; therefore, limiting the quantity of data transmitted may reduce their utility. Transmitting a big amount of data is definitely more power-expensive, but may results in a more accurate analysis of data. For this reason the authors have decided to do not process the sampled acceleration values before transmitting them.

Furthermore, in contrast to what has been done in other studies [23,24], it is important to notice that no frequency range reductions were applied to the accelerometer itself or to the board to artificially improve the default resolution R. Accelerometer resolution can be evaluated using the following equation:

(1) $$R=N\sqrt{1.6BW}$$

where N is the power spectral density noise in μm·s^−2^·Hz^−0.5^ and BW the bandwidth of the sensor. Equation (1) shows that the accelerometer bandwidth will determine the measurement resolution. Thus, applying a low-pass filter on the output of the accelerometer's sensing axis, it is possible to reduce the bandwidth of the accelerometer and improve the default resolution R.

Systems proposed in previous studies adopted this solution to improve the sensing capability. Many high-sensitivity MEMS-based accelerometers are characterized by a quite large bandwidth (in the order of 10^2^ to 10^3^ Hz). Such a large bandwidth is too wide for traditional SHM problems, which are characterized by very low-frequency. Consequently, not using the whole bandwidth of the sensor makes sense to obtain a better resolution of the sampled data. In this study, the authors were interested in testing the sensor's effective capability. No signal conditioning section is embedded in the system. Once the effective resolution of the MEMS accelerometer system is evaluated, further studies will focus on the design of that section, for narrowing the bandwidth of the accelerometer and improving its resolution. In addition, since the prototype presented in this study is a multi-purpose one, applying a bandwidth reduction may limit the applications of the MEMS accelerometer system.

### 2.1. Architecture of the Transmitter Board

The main feature of the transmitter board is to measure acceleration and wirelessly transmit the signal to the receiver board. In a conventional MEMS accelerometer board, ADC is employed to convert the sensor's analog signal to digital signal for wireless transmission. ADCs installed on the first released sensor boards had a 10-bit resolution, while next generations of boards were instrumented with 16-bit resolution ADCs [18,23,25]. This is still inadequate for high-fidelity measurement of low-frequency, low-amplitude dynamic signals. For example, the MEMS sensor selected in this study, the SF1600 accelerometer, should be matched with a 24-bit ADC to achieve the designed performance [26]. In order to address this difficulty, the authors designed an analog, frequency-modulated wireless signal transmission system. Converting the sensor output voltage to a frequency signal through a V/F converter, and then wirelessly transmitting the frequency-modulated signal will not affect the accuracy of sensor signal.

This is definitely the main feature of the board. This decision may seem to go against the grain of today's board design idea. It implies transmission of a larger amount of data if compared with its digital counterpart; yet, at the same time, it allows for more accurate and reliable data in a particular frequency range, which is the final aim for which this board has been built. This technique is quite slow, but operating with high-sampling rate devices (order of MHz) and a narrow sensor bandwidth (0 to 1.5 kHz), it is possible to overcome this limitation [27]. Furthermore, the choice of this type of converter, because sensor output has low amplitude and low frequency, permits a signal, which is more immune to noise [12]. In the authors' intent, this peculiarity may allow the system to avoid nullifying accelerometer characteristics as other ADCs can do.

**Figure 2.**
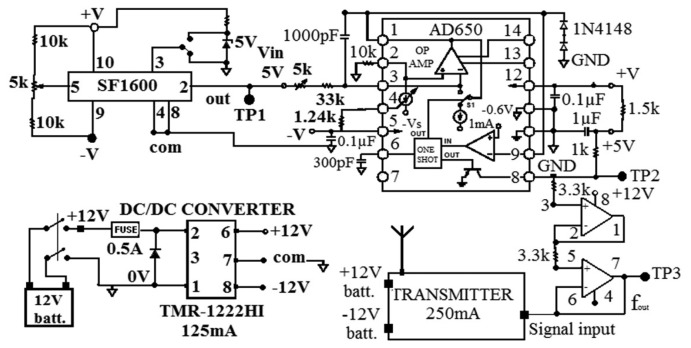
Circuit diagram of accelerometer transmitter board.

The architecture of the transmitter board is simple. The analog sensor output signal is first amplified and then converted to pulses through the V/F converter. It produces a pulse whose frequency is proportional to the signal's voltage value. The frequency modulation enables a wireless transmission immune to noise [28,29], which is particularly advantageous for transmitting low-amplitude, low-frequency vibration signals. A high-frequency V/F converter in the order of MHz is employed, considering the sensor's frequency range relevant to the targeted applications [29]. The V/F converter chip AD650 is selected to convert the sensor output to a sequence of pulses up to 100 kHz [30]. The 0 V sensor output is converted to 50 kHz. Figure 2 shows the circuit diagram of the transmitter board.

As shown in Figure 2, alongside the V/F converter, there are a variable resistor section (connected to the pin number five of the SF1600 and referred as offset trimmer) and a DC-to-DC (DC/DC) converter. Since MEMS accelerometers can be used to measure tilt angles as well, the variable resistor section was designed to remove DC signals coupled to gravity of the pendulum inside the accelerometer associated with the tilt angle. The section is made of two 10 kΩ resistors and a 5 kΩ multi-turn trimmer potentiometer deployed in series. It permits to correct the sensor's output tension value to remove the DC signals associated with the sensor orientation. On the other hand, the DC/DC converter, TMR3-1222HI [31], is used to provide a stable 12 V power supply to compensate for the gradual decrease in the battery power output over time. The diagram in Figure 2 also shows a signal amplification section for the signal coming out from the V/F before being sent to the RF transmitter. This signal amplifier addresses a major problem in vibration data transmission: amplitude-decreasing phenomenon in the low-frequency range [32].

**Figure 3.**
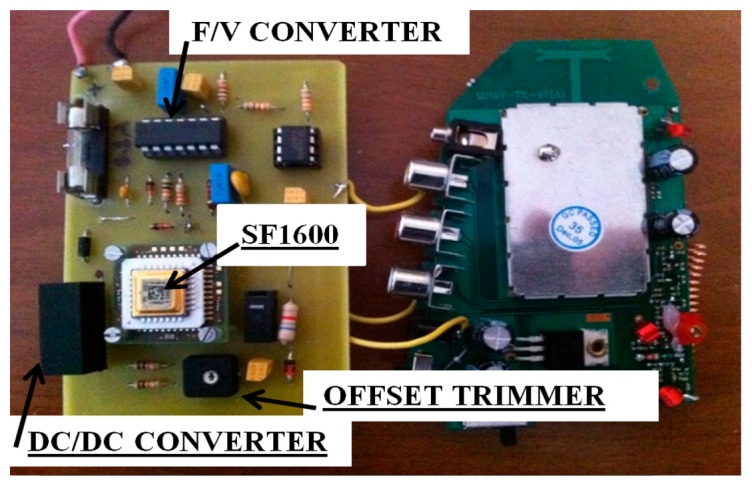
Prototype of the transmitter board.

The transmitting section consists of a low-cost 440 MHz, 4-channel antenna, each with a bandwidth of 5 MHz. Its theoretical transmission range is approximately 300 m in free field conditions. Figure 3 shows a prototype of the sensor board made in this study. This is the first prototype built for testing purposes and miniaturization will be carried out to reduce the size of the circuit to a few square centimeters in the future.

### 2.2. Architecture of the Receiver Board

The main feature of the receiver board is to collect the wirelessly-transmitted data and demodulate the signal. Analogously to the transmitter board, the receiver board is equipped with a 5 MHz, 4-channel, omnidirectional antenna, tuned to the same frequency as the transmitter board. The radio signal is not encrypted. In addition, a low-threshold discriminator is embedded in the board.

**Figure 4.**
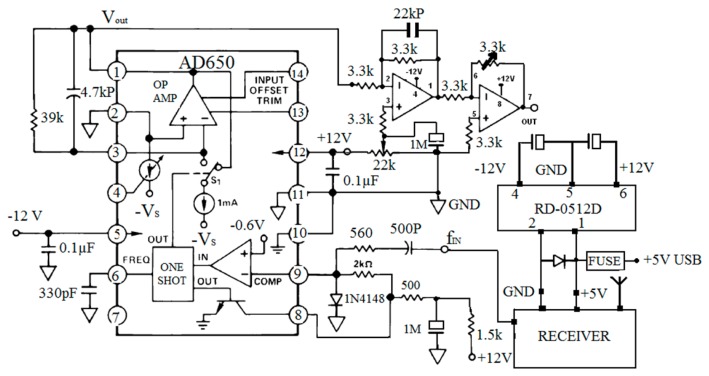
Circuit diagram of the receiver board.

**Figure 5.**
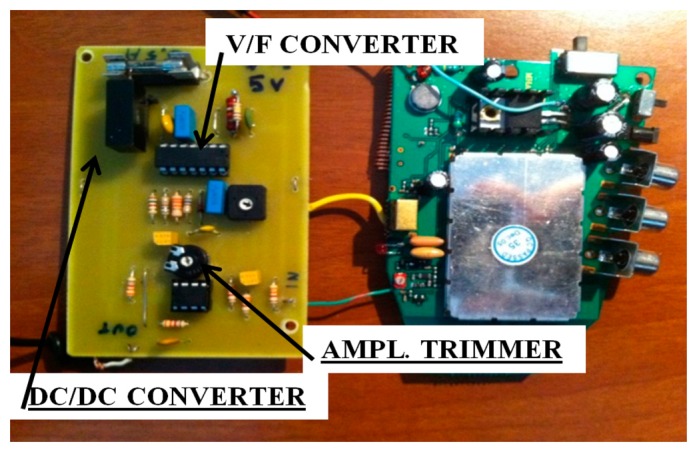
Prototype of the receiver board.

In the receiver board, the 0 to 100 kHz pulses received by the antenna are sent to a F/V converter to be reconverted to the original sensor signal analog voltage. The F/V converter is also an AD650 model, the same as the one used in the transmitter board. The obtained analog voltage is then amplified and normalized in the range 0–10 V through an amplification section, in which a 3.3 kΩ multi-turn trimmer potentiometer is deployed. The sensor data can be further acquired through an external data acquisition device. Figures 4 and 5 show the circuit diagram and the prototype of the receiver board made in this study.

A DC/DC converter model RD-0512D was installed on the receiver board [33]. The transmitter and receiver antennas work with a ±12 V tension. The installed DC/DC converter has been selected because it can convert an input voltage of ±5 V to ±12 V. This equipment, allows powering the receiver board using the Universal Serial Bus (USB) port of any laptop or computer, which is convenient for in-field measurements. USBs supply a voltage of ±5 V, which by means of the DC/DC converter is transformed in a voltage of ±12 V, suitable for the antenna to work. The decision to use a 12 V battery depends on the RF system only. In real-world engineering practices, the dimensions of this type of batteries could be a serious issue for the MEMS accelerometer system deployment. As shown in the following paragraphs, since the authors consider this RF antenna as temporary, once a different transmitter-receiver apparatus is selected, it will be possible using a different and smaller cell to power the device.

## 3. Evaluation Tests

To evaluate the capabilities of the prototype wireless MEMS accelerometer system, several tests were carried out. They include tests of the wireless transmission distance in two different conditions and the wireless transmission performance under diminishing battery power. Furthermore, static tests using a tilting machine and dynamic tests using electromagnetic shakers were carried out. In these tests, the wirelessly acquired MEMS sensor signals are compared with those given by a traditional wired sensor as reference. In addition, the wireless MEMS accelerometer system was deployed on a pipe to measure and analyze its flow-induced vibration.

### 3.1. Calibration Test of the Accelerometer System

**Figure 6.**
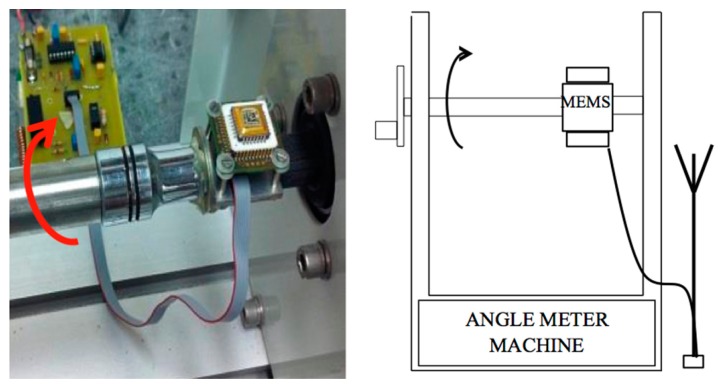
Setup for the static calibration test.

The wireless sensor system was first evaluated through a static calibration test. In it, the accelerometer was tilted to different angles and the wirelessly transmitted sensor output signals were measured. As shown in Figure 6, the MEMS accelerometer, fixed on a tilting machine, is rotated from to 0° through 360° at intervals of 5° ± 0.01°. The receiver board, connected to a laptop for DAQ, was placed one meter away to measure the wirelessly transmitted sensor output signal. For each inclination, a 10-minute measurement was made and the wirelessly transmitted sensor output signals were acquired with a 3 kHz sampling rate using an external DAQ device connected to the receiver board. A NI-USB 6009 with eight analog 24-bit input channels, and able to sample up to 48 kilo Samples per second (kS/s) was used in this study. The mean and standard deviation values were computed for each set of the 10-minute measurement data.

**Figure 7.**
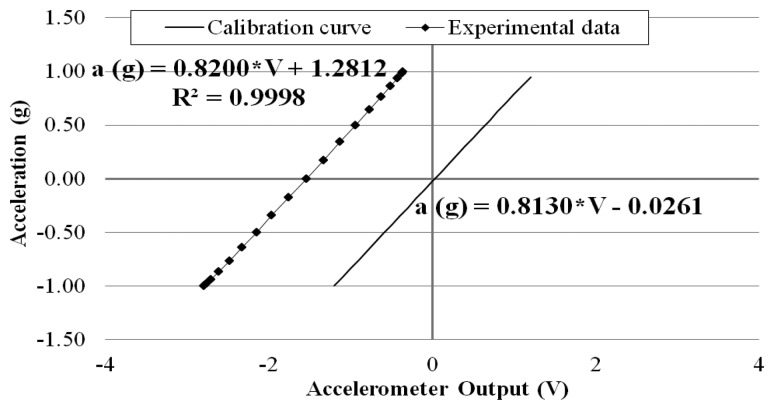
Results of accelerometer system calibration tests.

In Figure 7, for each inclination, the component of the gravitational acceleration—orthogonal to the accelerometer's pendulum mass—is plotted against the mean value of the measured accelerometer's output voltage. In all tests done, the standard deviation was always smaller than 0.009 mV, demonstrating the stability of the wirelessly transmitted sensor output data over the 10-minute measurement period.

The calibration test data shown in Figure 7 confirm the linear relation between the acceleration and the sensor output voltage with a high correlation of R^2^ = 0.9998. The measured calibration coefficient (*i.e.*, the slope of the measured line), 0.820 g/V, is highly close to the sensor-maker supplied 0.813 g/V [22]. The constant between the measured and the supplied lines (1.281 g), can be easily offset by adjusting the multi-turn trimmer potentiometer on the transmitter board without affecting the slope of the line.

### 3.2. Maximum Transmission Distance

**Figure 8.**
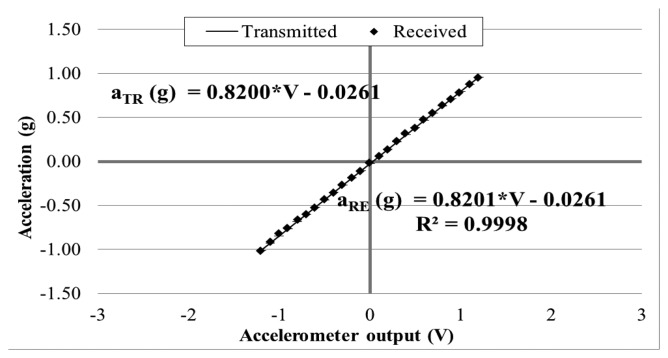
Calibration chart when receiver is 5 m away from transmitter.

**Figure 9.**
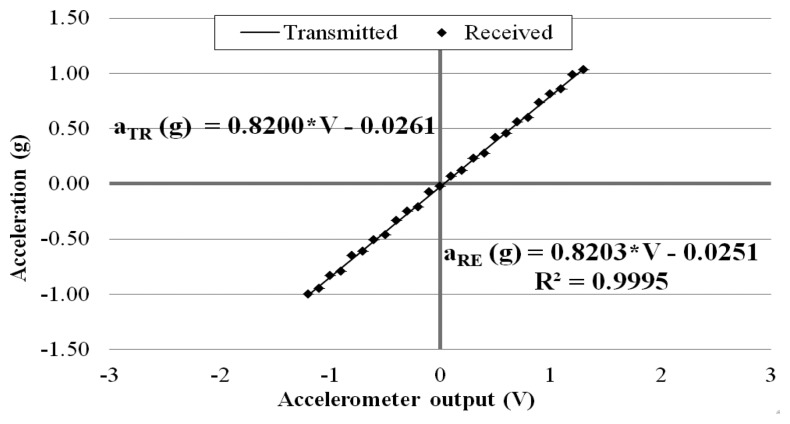
Calibration chart when receiver is 15 m away from transmitter.

**Figure 10.**
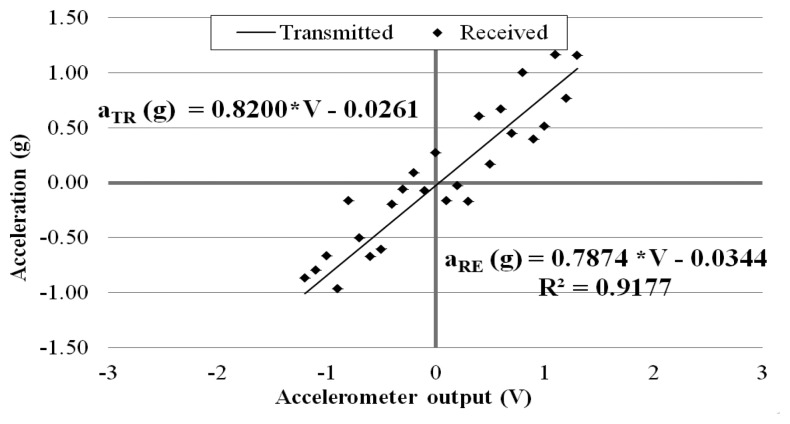
Calibration chart when receiver is 30 m away from transmitter.

In order to evaluate the wireless signal transmission capability of the prototype sensor system, the above calibration tests (as shown in Figure 6) were repeated by placing the receiver board to different distances away from the transmitter board: 5, 10, 15, 20, 25, and 30 m. This time, at each of the inclinations, the sensor output signal was recorded at the exit of the transmitter board and at the entrance of the receiver board placed away. Again, data were acquired for 10 min at 3 kHz through the external DAQ device. Figures 8, 9 and 10 plot the accelerometer output signals sampled at the output section of the transmitter board (Transmitted) versus those sampled at the receiver board (Received) placed 5, 15, and 30 m away from the transmitter board.

It is observed that the quality of the wirelessly transmitted signal decreases, as the distance between the transmitter and receiver increases. When the distance is equal to 5 m, an excellent agreement is observed between the data directly measured at the accelerometer output and those obtained at the receiver board, with a coefficient of correlation R^2^ = 0.9998. On the other hand, when the two boards are distanced 30 m apart, the wirelessly transmitted signals become less accurate. This is due to the decreased signal strength (and, thus, decreased signal-to-noise ratio) over the longer distance.

**Figure 11.**
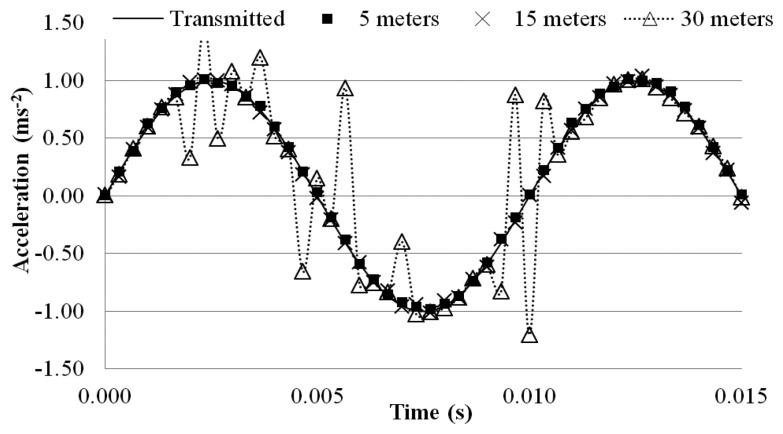
Time histories of wirelessly transmitted signal (Indoor).

**Figure 12.**
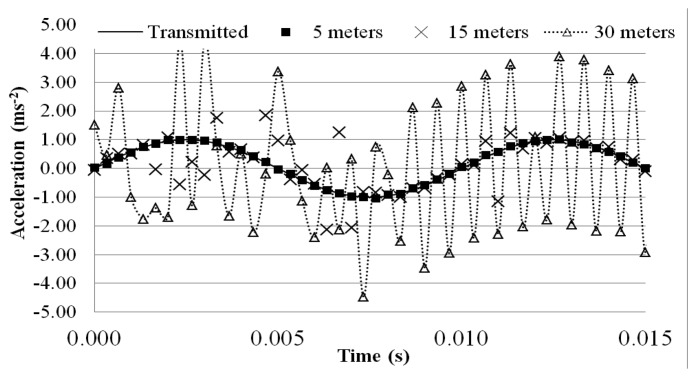
Time histories of wirelessly transmitted signal (Outdoor).

This problem can be observed when a dynamic test is carried out. The MEMS accelerometer was excited with a 100 Hz sinusoidal wave, while the receiver board was placed to different distances away from the transmitter board to measure the wirelessly transmitted sensor output signal. The test was carried out in two different conditions: (a) indoor (controlled environment in the basement of a laboratory with no wireless and RF sources different from the MEMS based system) and (b) outdoors (noisy urban campus environment characterized by a widespread use of RF systems, such as radio-communications, Wi-Fi networks, mobiles, *etc.*). For each distance, a 10-min measurement was made and the wirelessly transmitted sensor output signals were acquired with a 3 kHz sampling rate using the external DAQ device connected to the receiver board. To evaluate the quality of the transmitted sinusoidal signal, a relative error *ε_r_* (%) was computed using the following equation:

(2) $${ɛ}_{r}\left(\%\right)=\frac{1}{N}\sum_{i}^{N}{\left(\left|\frac{x_{\text{tra}}\left(t\right)-x_{\text{rec}}\left(t\right)}{x_{\text{tra}}\left(t\right)}\right|\right)}_{i}*100$$

where *x_tra_* (t) is the acceleration value measured in the output section of the transmitter board at time t, *x_rec_* (t) is value recorded at the receiver board, and N is the total number of data points measured during the 10-minutes period. Tables 2 and 3 tabulate the relative error with the distance evaluated over the 10-min measurement period. Figures 11 and 12 plot a segment of the time histories of the wirelessly transmitted sensor output signals measured at the receiver placed at the different distances, indoor and outdoor.

In indoor conditions, the signal remains stable for distances between the two boards less than 25 m, whereas the signal is distorted when the distance is 30 m, as confirmed by the relative error soaring from 0.42% to 7.26%. The theoretical transmission range of the antenna is nearly 300 m in free-field conditions, but the tests show that the effective distance drops by nearly 90%, and the performance further deteriorates in the outdoor environment, as shown in Table 3 and Figure 12. Relative errors soar from 1.08% at the 5-meter distance to 9.17% and 18.67%, respectively, at the 15-meter and 30-meter distance of the receiver board.

Obviously the maximum transmission distance depends on the surrounding environment and the interference with the surrounding RF noise causes significant concerns about the quality of signals. Previous studies have also highlighted the same interference issues with commercial wireless sensors caused by mobile phone traffic or, worse, communication between transmitter and receiver board lock-ups [14]. At present, experiments demonstrate that the MEMS accelerometer system cannot be used for carrying out measurements on area having a wide extension. It is reasonable to think that a more effective RF transmitter-receiver apparatus may solve problems related to signal deterioration over distance. Further developments of this study will involve the selection of a RF system more suitable for SHM purpose over a wider area. If this solution should fail, then the research may be oriented to select a more effective on-board conversion system.

### 3.3. Effect of Battery Residual Charge

Previous studies suggest that an unstable battery power supply voltage would affect the performance of a wireless sensor system [34]. To ensure a stable 12 V power supply even when the battery power starts to run out, the DC/DC converter is incorporated in the transmitter board design in this study. In order to investigate the effectiveness of the DC/DC converter, tests were carried out to measure the sensor outputs under power supply voltages less than 12 V.

In the test the transmitter board was powered using a programmable AC/DC converter and the supplied voltage was dropped from 12.5 V to 6 V at the 0.1 V interval. This to simulate a progressive battery cell exhaustion. The transmitter board was placed horizontally at rest (*i.e.*, 0° inclination). At each power supply voltage, the accelerometer output wirelessly transmitted to the receiver board was measured for 10 min, and the data were acquired at 3 kHz sampling rate by the external DAQ device connected to the receiver board.

**Figure 13.**
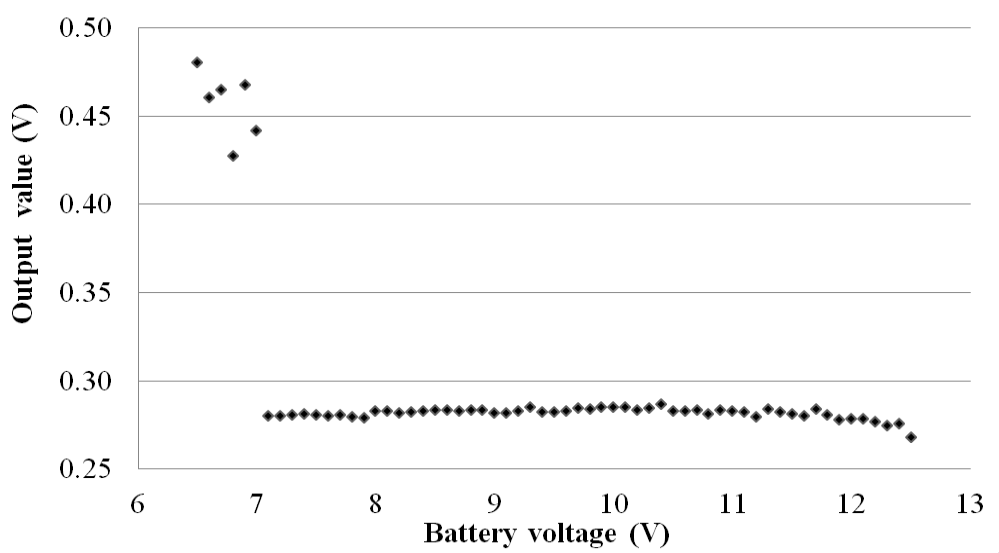
Effect of battery residual charge on sensor output.

Figure 13 plots the means of the measured sensor outputs, recorded from the receiver board, corresponding to the different power supply voltage. In the range 12.5 V–7 V, the output values are constant and remain stable. This means that the decreasing battery voltage does not influence the sensor output. When the battery power is below 7 V, however, the sensor output data become unstable and no longer reliable. Realistically, a 12 V battery cell will stop working when its residual charge drops to 9 V.

To be rigorous, analyzing the measurements in Figure 13, the maximum difference in the output values between 12 V–7 V is equal to 8 mV (Output value_max_ = 0.278 V, at 10.4 V and Output value_min_ = 0.270 V, at 11.2 V), which correspond to a fluctuation of 6.34 × 10^−2^ m·s^−2^. If the same data are recorded in the output section of the transmitter board, the maximum change is smaller than 1 mV, which corresponds to a fluctuation of 8.04 × 10^−3^ m·s^−2^.

It is possible to conclude by saying that the progressive battery cell exhaustion does not affect the performance of the wireless MEMS accelerometer system as in previous analyzed systems [34]. The DC/DC converter is effective in compensating the drop of the battery charge below 12 V; nevertheless, this experiment demonstrates again that the selected RF system may alter the recorded data.

### 3.4. Comparative Tests on Shaking Tables

In order to evaluate the performance of the wireless MEMS accelerometer system in measuring low-frequency, low-amplitude vibration, shaking table tests were carried out using a wired piezoelectric accelerometer (model 393B04 by PCB Piezotronics Inc. [35]) as a reference sensor. The sensors were excited by sinusoidal and periodic motion of the table, and the wirelessly transmitted sensor output signals were compared, in time and frequency domains, with those recorded by the reference sensor.

**Figure 14.**
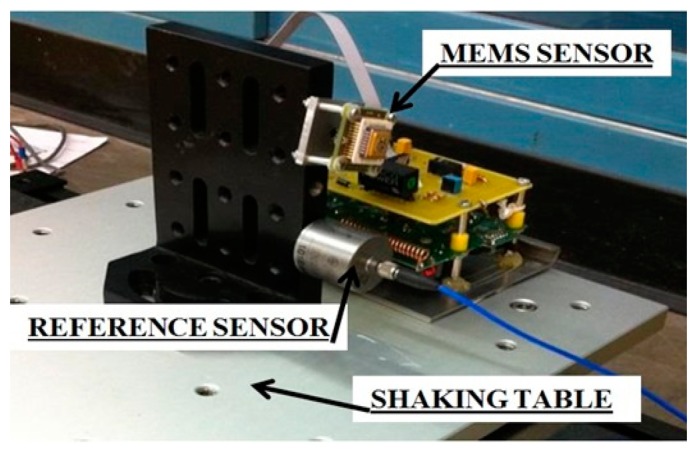
Setup for the shaking table test (sinusoidal wave input).

Figure 14 shows the wireless MEMS accelerometer and the wired piezoelectric reference sensor fixed on an electromagnetic shaking table. The table was excited with 5, 2, 1, 0.5, and 0.2 Hz sinusoidal waves. For each frequency, a 5-min measurement was made and the wirelessly transmitted sensor output signals were acquired with a 100 Hz sampling rate using the external DAQ device connected to the receiver board and the reference sensor. In other words, data from both of the sensors were acquired by the same DAQ system. The receiver board was deployed 5 m away from the transmitter board.

**Figure 15.**
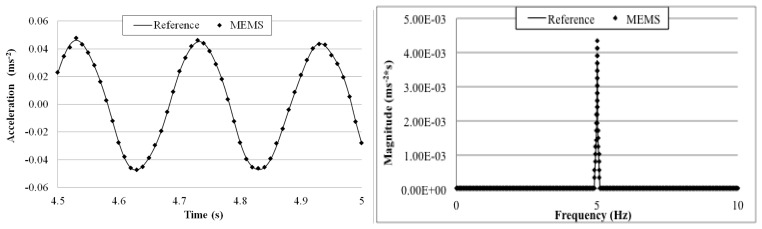
Comparison of measurements by the two Sensors in time and frequency domains (5 Hz sinusoidal excitation).

**Figure 16.**
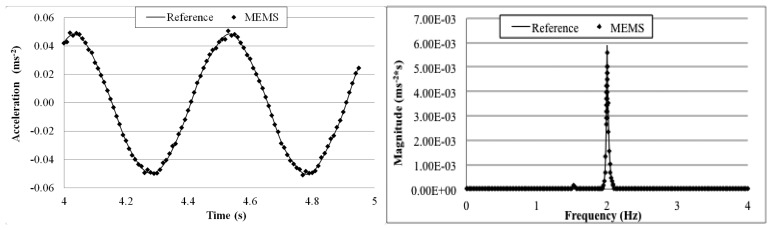
Comparison of measurements by the two sensors in time and frequency domains (2 Hz sinusoidal excitation).

**Figure 17.**
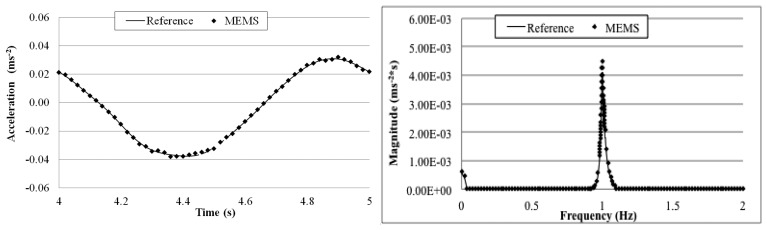
Comparison of measurements by the two sensors in time and frequency domains (1 Hz sinusoidal excitation).

**Figure 18.**
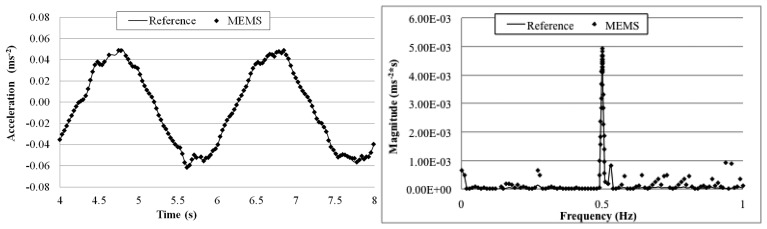
Comparison of measurements by the two sensors in time and frequency domains (0.5 Hz sinusoidal excitation).

**Figure 19.**
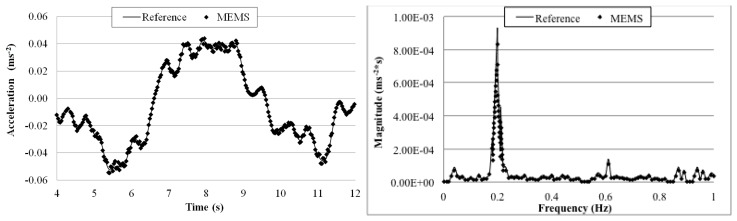
Comparison of measurements by the two sensors in time and frequency domains (0.2 Hz sinusoidal excitation).

Figures 15, 16, 17, 18 and 19 plot the recorded time histories and their corresponding Fast Fourier Transforms (FFT). A good agreement is observed between the data measured by the reference sensor and those by the MEMS sensor and obtained at the receiver board.

The measurement error of the MEMS sensor in comparison with the reference sensor was computed using the following equation and is listed in Table 4.

(3) $${ɛ}_{r}\left(\%\right)=\frac{1}{N}\sum_{i}^{N}{\left(\left|\frac{a_{\text{ref}}\left(t\right)-a_{\text{MEMS}}\left(t\right)}{a_{\text{ref}}\left(t\right)}\right|\right)}_{i}*100$$

where *a_ref_* (t) is the acceleration value measured with the reference sensor at time t, *a_MEMS_* (t) is value recorded with the wireless MEMS accelerometer, and N is the total number of data points measured.

From the measured acceleration time history and FFT plots in Figures 16, 17, 18 and 19 and the measurement errors quantified in Table 4, it is observed that the measurement error increases as the vibration frequency decreases. Overall the relative error is less than 1%, and, thus, the MEMS sensor achieved performance comparable to that of the reference sensor, demonstrating its capability in measuring low-frequency vibration, with frequency as low as 0.2 Hz with a suitable accuracy for normal engineering practices.

**Figure 20.**
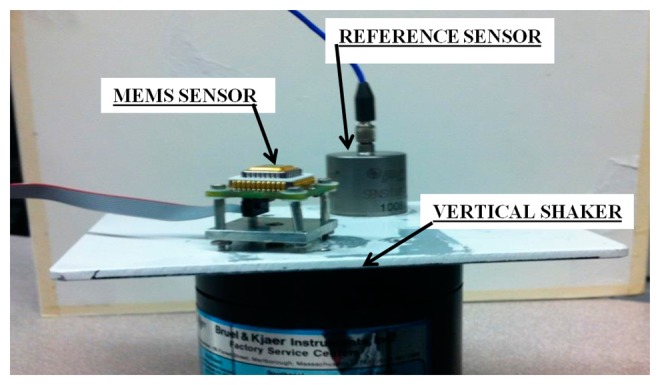
Setup for the shaking table test (periodic waves input).

Furthermore, the two sensors were fixed on a vertical shaker as shown in Figure 20, and the system was excited with periodic vibrations of 5, 2, 1, and 0.5 Hz. For each frequency, a 5-min measurement was made and the transmitted sensor output signals were acquired with a 100 Hz sampling rate using the external DAQ device connected to the receiver board (deployed 5 m away from the transmitter board) and the reference sensor.

**Figure 21.**
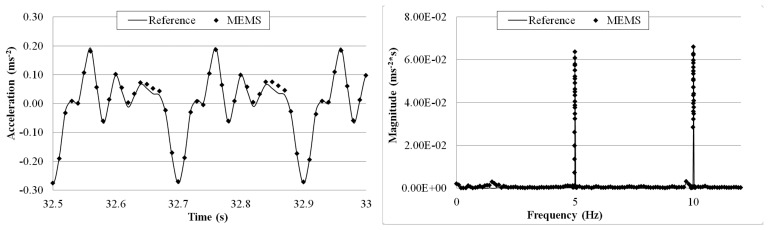
Comparison of measurements by the two sensors in time and frequency domains (5 Hz Periodic Wave Excitation).

**Figure 22.**
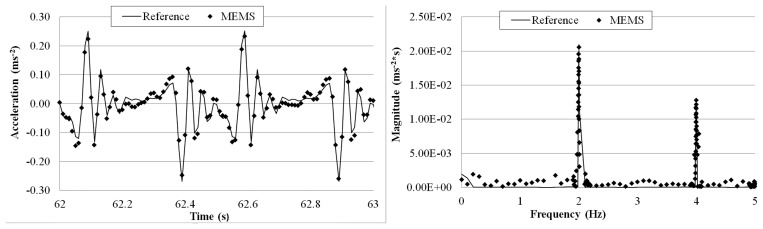
Comparison of measurements by the two sensors in time and frequency domains (2 Hz Periodic Wave Excitation).

**Figure 23.**
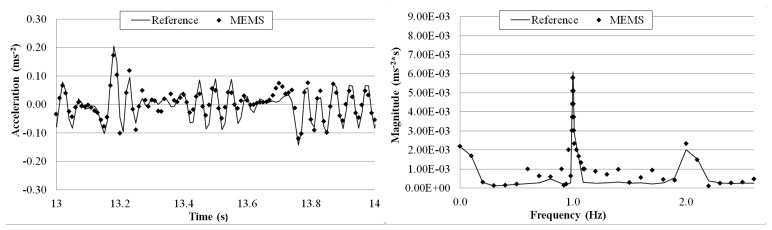
Comparison of measurements by the two sensors in time and frequency domains (1 Hz Periodic Wave Excitation).

**Figure 24.**
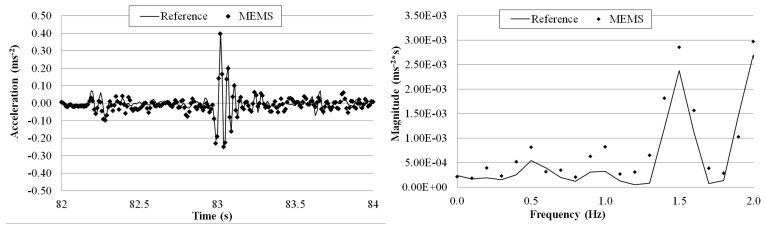
Comparison of measurements by the two sensors in time and frequency domains (0.5 Hz Periodic Wave Excitation).

The goal of this test is to evaluate the MEMS accelerometer system when more complex waves excite the structure on which the sensor is mounted. Figures 21, 22, 23 and 24 plot a detail of the recorded time histories and the corresponding frequency domain analyses calculated using a FFT analysis. Again, an excellent agreement between the data measured with the two sensors is observed. Even for vibration having frequency lower than 1 Hz (the value suggested by Directive as lower limit for detecting acceleration instead of displacement [36]).

Overall the measurements by the two sensors agree well with each other. The relative error of the MEMS accelerometer in comparison with the reference sensor is calculated using Equation (2) and the results, are shown in Table 5. Similar to the sinusoidal excitations, the measurement error increases as the frequency of the periodic vibration decreases. From the FFT plots in Figures 21, 22, 23 and 24, it is observed that the MEMS accelerometer system can identify the first frequency of the vibration and its high frequency harmonics as well.

### 3.5. Comparative Tests on Flow-Loop Pipeline

**Figure 25.**
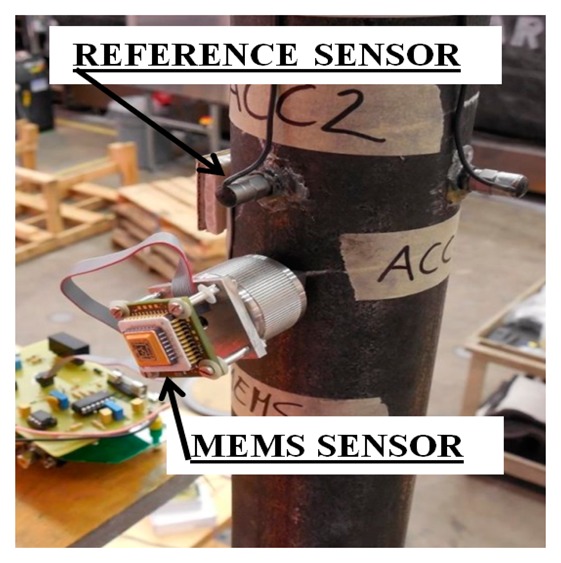
Setup for flow-loop pipeline test.

Finally, the wireless MEMS accelerometer system was used to measure oil flow-induced vibration of a black-steel flow-loop pipeline, and its performance was compared with a reference sensor. The set-up shown in Figure 25 is a special lab-scale model for testing the dynamic behavior of the flow-loop pipeline under pressure and temperature changes. Figure 25 also shows the two sensors used, the MEMS accelerometer and a reference accelerometer (W352C67 manufactured by PCB Piezotronics Inc. [37]) fixed next to each other on the pipe. The objective of the tests is to demonstrate the efficacy of the system in monitoring vibration of the model of a real engineering structure.

**Figure 26.**
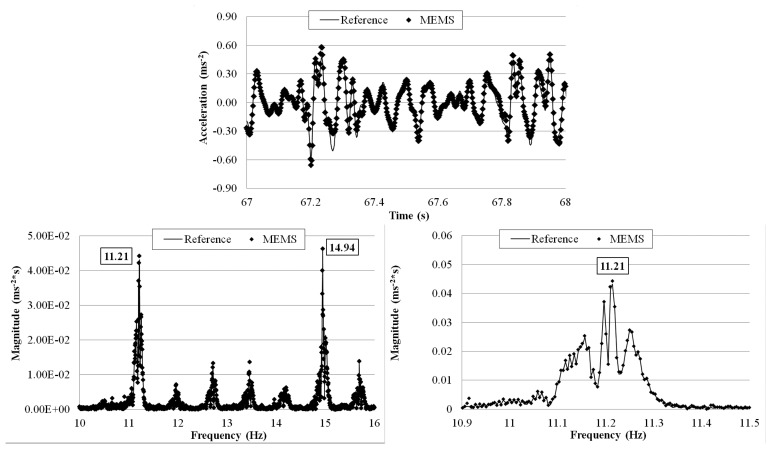
Comparison of measurements by the two sensors in time and frequency domains (8.00 × 10^−4^ m^3^·s^−1^ flow rate).

**Figure 27.**
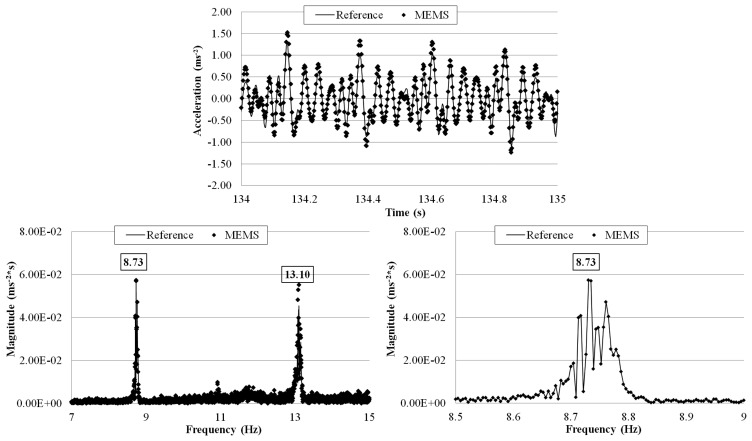
Comparison of measurements by the two sensors in time and frequency domains (1.35 × 10^−3^ m^3^·s^−1^ flow rate).

**Figure 28.**
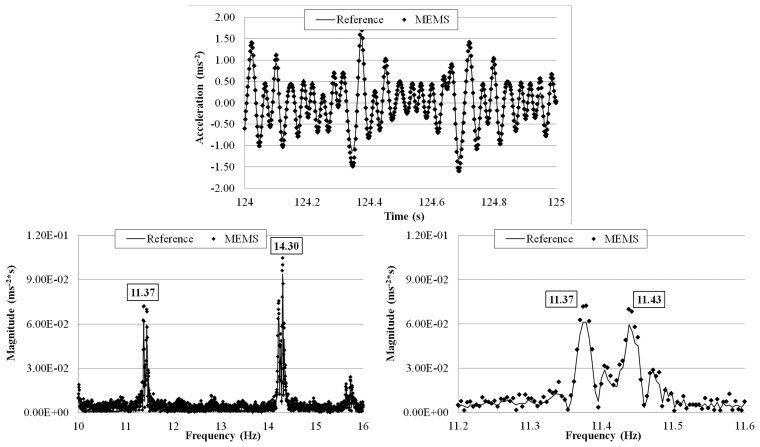
Comparison of measurements by the two sensors in time and frequency domains (2.00 × 10^−3^ m^3^·s^−1^ flow rate).

The tests were run with different flow rates: 8.00 × 10^−4^, 1.35 × 10^−3^, and 2.00 × 10^−3^ m^3^·s^−1^ and for each of them a 10 min measurement was made. The wirelessly transmitted sensor output signals were acquired with a 1 kHz sampling rate using the external DAQ device connected to the receiver board and the reference sensor. The receiver board (not visible in Figure 25) was deployed 5 m away from the transmitter board. Figures 26, 27 and 28 plot a segment of the time history measured by the two sensors under each of the three flow rates, together with its correspondent frequency response by FFT. The third plot in each of the figures shows a zoomed frequency response around the first mode frequency.

For all the three flow rates there is an excellent agreement, in both time and frequency domains, between the measurements by the MEMS accelerometer system and the reference sensor. Again, the relative error of the MEMS accelerometer in comparison with the reference sensor is calculated using Equation (3) and the results are shown in Table 6. The small errors are comparable with those computed in the shaking table tests. Consistent with the observations made in the shaking table tests, the measurement error increases as the first mode frequency decreases from 11.37 Hz (under the 2.00 × 10^−3^ m^3^·s^−1^ flow rate) to 8.37 Hz (under the 1.35 × 10^−3^ m^3^·s^−1^flow rate). Furthermore, the frequency response plots show the capability of the wireless MEMS accelerometer in measuring the closely coupled modes of vibration.

## 4. Conclusions

Wireless MEMS sensors suffer from low measurement accuracy when being applied to monitor low-amplitude ambient vibration of large-sized civil engineering structures that often have low natural frequencies. To overcome this problem, this paper presents a new wireless MEMS accelerometer system using low-cost, frequency-modulated analog RF transmission. A prototype system was built and extensive tests, including shaking table tests and pipe vibration measurements, were carried out to evaluate its performances in comparison with a high-performance, wired reference sensor. Comparing the measurements by the wireless MEMS sensor system and the reference sensor in both time and frequency domains, the test results demonstrated the accuracy of the wireless MEMS accelerometer system in measuring vibration relevant to civil engineering structures, including those of low frequency (up to 0.2 Hz) and low amplitude (in the order of 10^−1^ m·s^−2^). The measurement errors were less than 2%.

In addition, tests were carried out to confirm the stable performance of the wireless MEMS accelerometer system under draining battery power supply. The wireless signal transmission distance was also evaluated in both indoor and outdoor environments, which revealed a need for a future study to reduce the RF interferences over long-distance wireless transmission.

## Figures and Tables

**Table 1.** Micro Electro-Mechanical System (MEMS) accelerometer specifications.

**Quantity**	**Units**	**Typical Value**
Linear output range	m·s^−2^	±29
DC bias	m·s^−2^	±1.96
Sensitivity	V·m^−1^·s^2^	0.12 ± 0.01
Dynamic range (0.1/100 Hz)	dB	117
Output noise (10/100 Hz)	M·m·s^−2^ Hz^−0.5^	2.90
Frequency response (sensitivity ±3 dB)	Hz	0 to 1500

**Table 2.** Relative error of wirelessly transmitted signal (Indoor).

**Distance**	**Relative Error *ε_r_***
(m)	(%)
5	0.41
10	0.42
15	0.42
20	0.45
25	0.42
30	7.26

**Table 3.** Relative error of wirelessly transmitted signal (Outdoor).

**Distance**	**Relative Error *ε_r_***
(m)	(%)
5	1.08
10	1.46
15	9.17
20	12.34
25	16.29
30	18.67

**Table 4.** Relative error of MEMS accelerometer.

**Frequency**	**Relative Error *ε_r_***
(Hz)	(%)
5	0.29
2	0.53
1	0.53
0.5	0.84
0.2	0.99

**Table 5.** Relative error of MEMS accelerometer.

**Frequency**	**Relative Error ε_r_**
(Hz)	(%)
5	0.32
2	0.53
1	1.56
0.5	1.99

**Table 6.** Relative error of MEMS accelerometer.

**Flow Rate**	**Relative Error ε_r_**
(m^3^·s^−1^)	(%)
8.00 × 10^−4^	0.26
1.35 × 10^−3^	0.38
2.00 × 10^−3^	0.21
